# Dataset of distribution transformers for predictive maintenance

**DOI:** 10.1016/j.dib.2021.107454

**Published:** 2021-10-12

**Authors:** Diego-A Bravo M, Laura-I Alvarez Q, Carlos-A Lozano M

**Affiliations:** a Universidad del Cauca, Calle 5 Nro. 4-70, Popayán 190001, Colombia; b Universidad del Valle, Calle 13 Nro. 100-00, Santiago de Cali 760001, Colombia

**Keywords:** Distribution transformers, Predictive maintenance, Machine learning

## Abstract

In electricity sector is possible to collect large quantities of data that contain information on relevant processes and events that occur in a given period. It gives a knowledge of the different operation conditions of the electrical network and its components. Through the treatment and analysis of these data is possible to propose market, cost reduction, reduction of failures and repairs in machines and inventory decrease strategies. Grid operator can implement strategies to improve indicators of reliability and quality of service. From a maintenance point of view, the equipment operating time is a relevant aspect to identify and solve failures without service suspensions. This paper aims to show distribution transformers failures characteristics data using historical data collected by the grid operator (Compañia Energética de Occidente) at Cauca Department (Colombia), under the cooperation of the Universidad del Cauca and Universidad del Valle. The dataset could be helpful to researchers and data scientists who use machine learning to develop applications that help engineers in predictive maintenance.

## Specifications Table

**Table:** 

Subject	Electrical and Electronic Engineering
Specific subject area	Maintenance of distribution transformers
Type of data	Table
How data were acquired	Dataset was obtained through manual inspections at Cauca Department (Colombia) by Compañia Energética de Occidente.
Data format	Raw (*.xlsx format)
Parameters for data collection	Dataset of distribution transformers are connected to the operator’s network at voltage levels of 13.2 [kV] and 34.5 [kV], located in rural and urban areas at Cauca Department (Colombia).
Description of data collection	Dataset contains 16.000 electric power distribution transformers from Cauca Department (Colombia). They are distributed in rural and urban areas of 42 municipalities. The information covers 2019 and 2020 years, has 6 categorical variables and 5 continuous variables. First ones correspond to: location, self-protected, removable connector, criticality according to ceraunic level, client and installation type. Second ones are transformer power, burn rate, users number, unsupplied electricity and secondary lines length.
Data source location	Institution: Universidad del Cauca
	City/Region: Popayán, Cauca
	Country: Colombia
Data accessibility	Repository name: Mendeley Data
	Data identification number: 10.17632/yzyj46xpmy.4
	Direct URL to data: https://data.mendeley.com/datasets/yzyj46xpmy/4

## Value of the Data

• The data provide a collection of electric power distribution transformers characteristics at Cauca Department (Colombia).
• The dataset could be useful for researchers and data scientist studying the predictive maintenance by machine learning algorithms.
• The dataset are suitable for classification and regression models by machine learning algorithms.

## 1 Data Description

The dataset consists in two archives in Excel:

(i) Dataset_Year_2019.xlsx
(ii) Dataset_Year_2020.xlsx

Each file has 16 columns with the information of characteristics of distribution transformers at Cauca Department during 2019 and 2020 years, columns in order ascendent are:

A. **Location:** Binary variable that indicates the area in which the transformer is located: 1 if it is urban and 0 if it is rural.
B. **Power:** Transformer capacity in [kVA]. For transformers immersed in oil the standard IEC 76-1 eestablishes normal service conditions as altitude above sea level no higher than 1000 [m] and room temperature higher than $-25{\;}^{\circ }\text{C}$ and less than $40{\;}^{\circ }\text{C}$.
C. **Self-protection:** Binary variable that indicates if the transformer has a switch internally for low voltage protection: 1 if it is self-protected and 0 if it is not.
D. **Average earth discharge density DDT:** Variable in $\left[\text{Rays}/{\text{km}}^{2}\;\text{year}\right]$ is defined as the average number of lightning strikes per square kilometer in a year.
E. **Maximum ground discharge density DDT:** Variable in $\left[\text{Rays}/{\text{km}}^{2}\;\text{year}\right]$ is defined as the maximum number of lightning strikes per square kilometer in a year.
F. **Burning rate:** Variable based on the reliability of the system and calculated from the failure events recorded in the years of study. It is defined as the number of failures of a component per unit of recording time.
  (1) $$TQ=\frac{\text{Failures}}{\text{Time}}\;\left[\text{Failures}/\text{year}\right]$$
G. **Criticality according to previous study for ceramics level:** Binary variable product of the result of a previous study carried out for the distribution company by third parties: 1 if due to its geographical location it is at high risk and 0 otherwise, it does not present high risk.
H. **Removable connectors:** Binary variable that indicates if the transformer installation has removable medium voltage connectors to carry out interventions without the need to open from the disconnector immediately upstream: 1 if it has the removable connectors installed and 0 if it does not.
I. **Type of clients:** Categorical variable that indicates whether the transformer mainly feeds stratum 1, 2, 3, 4, 5, 6, commercial, industrial or official residential users.
J. **Number of users:** Integer variable that indicates how many customers the transformer in question is supplying the electric power service.
K. **Electric power not supplied EENS:** Variable based on the risk involved in the failure, represents the [kWh] that the distribution company stops selling when the transformer stops operating due to a failure event.
L. **Type of installation:** Categorical variable that indicates whether the installed transformer is in a cabin, in a H-type structure, if it has a macro with an anti-fraud net, if it is a pad mounted type, if it is in a simple pole-type structure, an anti-fraud net pole, a metal tower or others.
M. **Air network:** Binary variable that indicates whether the transformers low voltage network is of the aerial type: 1 if it is indeed and 0 otherwise.
N. **Circuit Queue:** Binary variable that indicates if the transformer is located within the medium voltage network at a terminal point of the circuit: 1 if it is in the queue and 0 if it is in a passing point.
O. **km of network LT:** Continuous variable that corresponds to the length in [km] of the low voltage transformer.
P. **Burned transformers:** Binary variable that indicates if the transformer has been burned during this year: 1 if it is burned and 0 otherwise.

## 2 Experimental Design, Materials and Methods

The dataset contains all distribution transformers connected to 13.2 kV and 34.5 kV voltage levels, located at Cauca Department rural and urban areas, owned by Compañia Energética de Occidente. It is necessary to emphasize that transformers of private property (third parties), the government, and anyone different to network operator are excluded. The universe is made up of 15.869 transformers that meet the operating context and the interests of the company in residential, commercial, industrial and official sectors.

The dataset was used for predicting the failure of distribution transformers is addressed using Machine Learning techniques, Alvarez [1]. From a Machine Learning point of view this is a binary classification problem. The predictive model obtained through this approach allows the construction of a predictive maintenance plan reducing operating costs and optimizing the resources assigned to the maintenance area of Compañía Energética de Occidente.

**Fig. 1:**
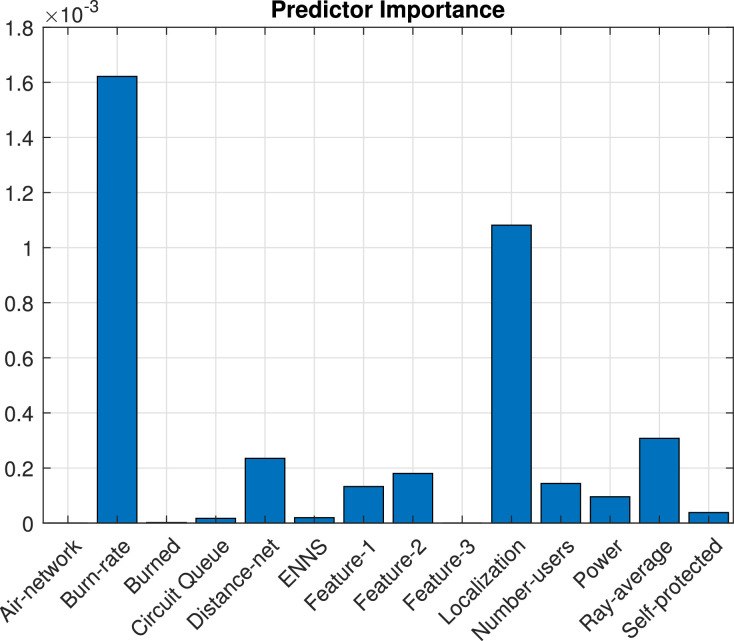
Variable importance measures from SVM classifier.

The binary classification algorithm used was the Support Vector Machine (SVM), which shows a lower percentage of error in the predictive capacity of failure in distribution transformers. The predictor variables $X_{i}$ of the training data set that most contribute to the predicted variable $Y_{i}$ of the model obtained through a binary classification with the SVM algorithm can be seen in Fig. 1.

Dataset is pre-filtered and does not contains data outliers or data missing, Bravo et al. [2].

## Ethics Statement

This work did not involve any human or animal subjects, nor data from social media platforms.

## CRediT Author Statement

All authors contributed equally in this work.

## Declaration of Competing Interest

The authors declare that they have no known competing financial interests or personal relationships which have, or could be perceived to have, influenced the work reported in this article.
